# Hospitalization for mental health related ambulatory care sensitive conditions: what are the trends for First Nations in British Columbia?

**DOI:** 10.1186/s12939-018-0860-7

**Published:** 2018-10-03

**Authors:** Josée G. Lavoie, Amanda Ward, Sabrina T. Wong, Naser Ibrahim, Darrien Morton, John D. O’Neil, Michael Green

**Affiliations:** 1 Department of Community Health Sciences, Ongomiizwin Research, University of Manitoba, Winnipeg, Canada; 2 First Nations Health Authority, Vancouver, BC Canada; 3 School of Nursing, University of British Columbia, Vancouver, Canada; 4 Department of Community Health Sciences, University of Manitoba, Winnipeg, Canada; 5 Faculty of Health sciences, Simon Fraser University, Burnaby, Canada; 6 Departments of Family Medicine and Community Health and Epidemiology, Queen’s University, Kingston, Canada

**Keywords:** Mental health, Indigenous health, Primary health care, ACSC, Nursing stations, First nations off-reserve

## Abstract

**Background:**

Indigenous peoples globally experience a disproportionate burden of mental illness due to forced policies and practices of colonization and cultural disruption. The objective of this study was to provide a baseline profile of hospitalization rates for mental health-related Ambulatory Care Sensitive Conditions among First-Nations living both on and off reserve in British Columbia, Canada, and explore the relationship between local access to health services and mental health-related hospitalization rates.

**Methods:**

A population-based time trend analysis of mental health-related Ambulatory Care Sensitive Conditions hospitalizations was conducted using de-identified administrative health data. The study population included all residents eligible under the universal British Columbia Medical Services Plan and living on and off First Nations reserves between 1994/95 and 2009/10. The definition of mental health-related Ambulatory Care Sensitive Conditions included mood disorders and schizophrenia, and three different change measures were used to operationalize avoidable hospitalizations: 1) rates of episodes of hospital care, 2) rates of length of stay, and 3) readmission rates. Data were analyzed using generalized estimating equations approach, controlling for age, sex, and socio-economic status, to account for change over time.

**Results:**

Our findings show that First Nations living on reserve have higher hospitalization rates for mental disorders compared to other British Columbia residents up until 2008. Those living off reserve had significantly higher hospitalization rates throughout the study period. On-reserve communities served by nursing stations had the lowest rates of hospitalization whereas communities with limited local services had the highest rates. Compared to other British Columbia residents, all First Nations have a shorter length of stay and lower readmission rates.

**Conclusions:**

This study suggests that despite reduced rates of hospitalization for mental-health related Ambulatory Care Sensitive Conditions over time for First Nations, gaps in mental health care still exist. We argue greater investments in primary mental health care are needed to support First Nations health. However, these efforts should place equal importance on prevention and the social determinants of health.

## Background

The poor mental health status of Indigenous peoples globally is a result of historical and ongoing legacies of colonization and systemic disadvantage manifest in state policies and service provisions, purportedly designed to promote or restore health [1]. In the Canadian context, past and ongoing Eurocentric policies have been implicated in rapid cultural change, forced assimilation, and dispossession. These policies have severed links between culture and identity and resulted in the criminalization and continued pathologization/problematization of cultural beliefs and practices. For example, the forced removal of Indigenous students from their families and communities to attend residential schools (1884–1996), where Indigenous languages and cultural expressions were banned, and where malnutrition and abuse were prevalent, has been implicated in multigenerational trauma [2–5]. Combined, these have gravely undermined Indigenous communities’ capacity to be self-determining [1, 6–10]. Multigenerational trauma has resulted in mental distress expressed through higher rates of posttraumatic stress disorder, depression, suicide, and substance use [8, 11]. While concerted efforts are being undertaken to ameliorate these alarming outcomes, disparities in mental health outcomes continue to persist between Indigenous and non-Indigenous populations in settler-colonial nations [12].

Primary health care (PHC) has been consistently heralded as a significant component of well-functioning health systems, leading to the promotion of population health and the prevention of disease, including mental health and illness [13]. Calls to action have been made to improve healthcare inequity through reforms over the administration and delivery of PHC services. These calls have been articulated for decades through documents such as the historic Declaration of Alma-Ata in 1978 and the World Health Organization’s 2008 report, *Primary Health Care: Now More than Ever* and the more recent Truth and Reconciliation Commission [13–15]. A key feature of PHC is that it seeks to deinstitutionalize care and instead locate services in the community. When mental health care (MHC) is integrated with PHC services, it can potentially offer patient/family-centric holistic care, address treatment gaps, reduce costs, promote human rights by mitigating stigmatization and social exclusion, enhance access to services closer to families and communities, and produce positive mental health outcomes [16].While a growing impetus to integrate MHC into PHC services is occurring globally, documented cases demonstrate that integration has been fraught with challenges. Challenges vary but are commonly characterized by fiscal constraints, organizational issues, availability of medical technologies and public health surveillance systems, and the cultural and technical competence of health professionals working with marginalized groups [16].

Among settler-colonial nations such as Australia, Canada, New Zealand and the United States, reforms propose to better align mental health services with PHC principles, in the hope of responding more effectively to the needs of Indigenous peoples [17–20], and to close the gaps that demarcate the shared experiences of health inequities reported for Indigenous peoples, when compared to their national counterparts. National rates of mental disorders among Indigenous peoples, which can result in self-medication and substance abuse, are not clearly known due to community variations, scarce non-standardized data, and the lack of an Indigenous identifier in national datasets. In Canada specifically, while a majority of First Nations report good mental health and balance, a significant proportion living within or outside First Nations communities experience distress or mental disorders and substance abuse at higher rates compared to the Canadian population [21, 22]. To address this issue, the Truth and Reconciliation Commission recommended the development of holistic Indigenous healing centres [15]. Indigenous nations have advocated for programs designed by Indigenous communities, and that reflect Indigenous perspectives on wellness and Indigenous treatment modalities [8, 23–25].

Estimates using symptomatic indicators have reported elevated levels of depression (18%) and alcohol use disorders (27%) among First Nations and Inuit peoples [26]. Despite many studies related to Indigenous mental health, a scarcity of studies quantifying and exploring the link between access to PHC services and mental health outcomes exist. To date, studies have not focused on mental health-related Ambulatory Care Sensitive Conditions (ACSC) specifically.

The purpose of this study was to document the rates of hospitalization for a selection of mental health-related conditions for First Nations in British Columbia (BC) living across different jurisdictional and geographical locations compared to rural and urban BC residents, and explore the relationship between local access to PHC services and mental health outcomes among First Nations communities. BC was chosen because it offers a unique context to explore the research objectives given the variability of community sizes, remoteness and therefore access to mental health services, and diversity of First Nations based on languages, cultures, histories, and local economies. Currently, there has also been no work examining the optimal complement of PHC services required in BC rural and remote communities. While studies conducted elsewhere have documented barriers in access to appropriate PHC services for Manitoba on-reserve residents, which are associated with higher hospitalization rates [27], it is unclear if this same pattern can be generalized to First Nations in BC. A key challenge facing BC First Nations communities is that they are relatively small, making access to PHC services much more challenging. As a result of a recent shift in governance over First Nations health in BC in 2013, generating empirical evidence to address these gaps in knowledge was identified as a significant research priority to inform and support mental health policy development and planning. These findings have relevance for international communities considering or undergoing PHC reforms to integrate MHC in response to the needs of Indigenous or rural communities.

### Conceptual framework: access to health care services

Inadequate access to health care services has long been conceptualized as a determinant of health, reproducing disparities or inequalities in health outcomes between and within populations [28]. While determinants of health are far-reaching to include genetics, lifestyle factors, socio-economic conditions, histories of oppression and physical environments [29–31], we employ this conceptual framework to explore the differences of mental health outcomes as a function of access to PHC between First Nations and other BC residents, among First Nations living on and adjacent to First Nation reserves, and off reserve in BC communities. BC population level health data shows that mortality rates from medically treatable illnesses among status First Nations were higher compared to other BC residents (1.5 per 10,000 vs. 0.3 per 10,000), potentially signaling gaps in access to PHC [32]. Our study focuses on mental health outcomes which we postulate can be improved by the provision of community PHC-based mental health services.

The need for PHC interventions can be defined by the capacity to benefit one’s health status from these interventions, which may occur in the form of enhancement, restoration, preservation or protection of an individual or community’s health [33]. For this study, we have expressed outcomes as hospitalization for mental health-related ACSC. Also known as avoidable or preventable hospitalizations, ACSC are defined as diseases or conditions that, if managed in a timely and efficient manner through PHC services, could prevent the onset of illness, control acute episodic conditions, and improve the management of chronic conditions [34]. Higher rates of ACSC suggest ineffective, unresponsive and/or barriers to access PHC services [35]. These proxy indicators have been widely accepted and validated as a measure of access to PHC services compared to self-reported survey data which may lack scientific rigour [35]. They are used by researchers and policy-makers for identifying gaps in the delivery of PHC and providing opportunities for targeting health care service interventions. To explore health disparities between First Nations and other Canadians and their link to PHC, other Canadian studies have successfully employed ACSC to suggest a need for investments in PHC in First Nations communities [27, 36, 37].

### Access to health care services

In BC, at the time that the data for this study was collected (1994/95 to 2009/10), the federal government funded a limited complement of PHC services on First Nations reserves. This system remains in place in all other Canadian provinces, but in 2013 health services for BC First Nations communities became the responsibility of the First Nations Health Authority, an organization mandated by First Nations Chiefs in BC to advance a shared vision of healthy, self-determining and vibrant BC First Nations, in full partnership with both federal and provincial governments, and Regional Health Authorities in BC [38, 39]. The service environment described below continues to create confusion around service provision to First Nations communities in other Canadian provinces, but in BC the BC First Nations Health Authority (FNHA) has undertaken a program of service integration that is progressively transforming service delivery. Nonetheless the results of this study are still important in BC for priority setting in PHC re-structuring, and to the rest of Canada for general implications in serviced arrangements.

The complement of services offered in each First Nation community varies based on community size, level of remoteness and geographical proximity to provincial services (see Table 1). This approach, which is based on recommendations put forth by a 1969 study which conceptualized the responsibility of federal government as complementary to those provided under provincial jurisdiction [40], failed to recognize the perpetually shifting jurisdictional complications between provincial and federal roles in relation to First Nations, and erroneously assumed that the provinces would eventually take responsibility for First Nations health [41]. Although access to provincial services has shifted over time (notably through the closure of rural hospitals or reduction in the number of hospital beds in the 1990s) and new programs have been added periodically to adjust for shifting needs (including the transition from infectious diseases to chronic conditions), the framework has remained fairly static. Studies have shown that First Nation communities with access to a broader complement of community-based PHC have better outcomes [27]. Still, other studies suggest that federal investments have failed to keep up with needs, resulting in local health services managing increasingly complex needs with dwindling resources [42, 43]. Local PHC services vary in provisions across communities based on available services, hours of operation, and proximity to provincial facilities (Table 1).

**Table 1:** Definition of the sample

Level of primary health care available on-reserve	Number of First Nation communities
Nursing Station	10
Health Centre	12
Health Station	47
No Facility	56

Although some First Nations communities have *no local access* to federally-funded PHC because of their proximity to provincial services. Communities considered to have only reasonable access to health care in nearby communities are typically served by an on-reserve *health station*. *Health stations* are staffed by part-time non-resident nurses and resident part-time community staff offering screening and prevention services only. *Health stations* are generally located in smaller communities with year-round road access to a provincial point of care located close by (family physician, rural hospital, or a nursing station). In contrast, *health centres* are located in communities where the closest provincial facility is 2 h away. Services are provided 5 days per week, including emergency care, screening and prevention services. However, recruitment and retention issues can result in lapses in the provision of services at health centres, and road access is at times limited to unpaved, logging roads. Finally, *nursing stations* provide screening, limited treatment, prevention services, emergency care and treatment services on a 24/7 basis by nurses with an expanded scope of practice. *Nursing stations* are often located in larger and more isolated communities that are fly-in only, or those served by roads that are operational only in the winter.

In regards to MHC services specifically, three main federal programs have been established to provided mental health counselling, funded through different mechanisms [44]. The Short-Term Crisis Intervention Mental Health Counselling (STCIMHC) provides for community support and counselling sessions at times of crisis (following suicides in a community, for example). Under this program, an individual can be eligible to up to a maximum of 15 one-hour sessions per mental health crisis over a 20-week period of from a counsellor approved by the First Nations and Inuit Health Branch of Health Canada (FNIHB), which funds on-reserve health services and approves qualified counselors (i.e. psychologists, social workers with clinical counselling orientation or mental health counsellors). All former Indian Residential School students and family members [see 2 for a detailed overview] can apply for cultural and/or emotional support or counselling under the Indian Residential Schools Resolution Health Support Program (IRS RHSP). In addition, all communities benefit from the National Native Alcohol and Drug Abuse Program (NNADAP), which provides primary prevention and support and coordinates referrals to residential treatment centres. Workers under these programs are generally community residents with variable levels of formal education. The program has historically been severely underfunded. Workers in most communities now provide support to families facing mental health and substance abuse challenges, with little additional training or support [43]. Finally, the National Aboriginal Youth Suicide Prevention Strategy (NAYSPS) supports youth-centred suicide prevention interventions.

First Nations living off reserve are expected to access health care services under provincial jurisdiction similar to all Canadians, as defined by the *Canada Health Act*, *1984* [45]. Health care services deemed medically necessary are funded through federal transfers and provincial taxes, and the province administers services regionally. Concurrent to the regionalization of BC’s health care system in 2002, MHC had been undergoing a process of deinstitutionalization since 1998 to institute regionalized mental health community-based programming [46]. This process, however, has occurred with little investment in community-based mental health services. Investments in First Nations-centric, culturally safe, trauma-informed mental health services that integrate Indigenous treatment modalities have not occurred.

Empirical studies examining the relationship between access to care for First Nations and health outcomes are limited. However, evidence suggests that access may not simply be a function of geographic accessibility but may be undermined by jurisdictional ambiguities, quality, transportation policies [27, 47] and variable responsiveness. For example, despite the close proximity to health services in urban settings, utilization of specialist care by First Nations remains low [48], suggesting that referrals are not being extended to First Nations to the same extent as to other Canadians, and/or that First Nations are reluctant to accessing specialists because of past histories of being discounted, racism and culturally unsafe care [49–51]. Findings from studies investigating access to care for both Indigenous and non-Indigenous rural populations suggest that non-physician health professionals and telehealth services may be a viable option to improve access to care, given the constraints of physician supply in rural and remote communities [27, 52, 53]. Finally, recent work by Kyoon-Achan and colleagues clearly shows that First Nations consider mental healthcare to be an important component of primary health care [54]. The same authors have also shown that hospitalization for ACSC mental health conditions we included in this study are lower in communities with better access to primary healthcare [55].

## Methods

### Project background

The *Closing the Gap* research project was initiated as a partnership between the BC First Nations Health Authority (FNHA), and Canadian university researchers from the University of Manitoba, the University of British Columbia, Simon Fraser University and Queens University. The project sought to provide evidence-based information detailing access to PHC among First Nations and rural and remote communities in BC by assessing models of care available in these communities using hospitalization rates for ACSC as a key indicator. The findings are intended to assist the FNHA in decision-making, priority setting and advocacy roles.

### Data sources and sample

The research sample includes all BC residents eligible under the provincial Health Services Insurance Plan and First Nations living on or off reserve. In BC, postal codes tend to cover larger areas and First Nations reserves are small. A very small number of communities cannot be uniquely identified by postal code. In this study, we were able to use the BC Medical Services Plan, which tracks payers of BC Health Premiums as a proxy indicator to identify First Nations. In the case of First Nations, the payer is the Federal health agency FNIHB. We were able to use this proxy indicator combining the six-digit postal code and premium payer to track First Nations individuals living on reserve in BC. Thus, using a combination of postal code and premium payer to identify First Nations also resulted in the inclusion of First Nations adjacent to the reserve, without including other residents in the analysis. Although these First Nations technically live off reserve, their proximity to a reserve often result in them using on-reserve service to meet their needs [43]. This approach is defendable since this study is concerned with access to services, instead of a stringent application of residence.

The hospitalization data used for this study were acquired through the Ministry of Health Services and the University of British Columbia (on behalf of Population Data BC) and included: (1) the Discharge Abstract Database (DAD), (2) Consolidation file, and (3) Medical Service Plan (MSP) data. DAD contains data on discharges, transfers, and deaths of inpatients and day surgery patients from acute care hospitals in BC. The Consolidation file contains demographic information such as age and sex, postal codes indicating location of residence, and registration data. MSP data offers information on medically necessary services provided by fee-for-service practitioners to individuals covered by the MSP, BC’s universal insurance program. Lastly, census data (1994–2010) contained demographic and ecological information (such as postal codes indicating location of residence). We had access to data from 1994 to 2010. For the “other urban” and “other rural” categories we only had access to data from 1999 to 2010.

Community information was obtained from a database based on information in the public domain, which contains six-digit postal code information for each on-reserve community and the level of care available on reserve (Table 1), information gathered from First Nations community profiles obtained from the Aboriginal Canada Portal and other public sources, and Indigenous and Northern Affairs Canada and FNIHB on-reserve population figures. Data were linked based on the needs of each individual study after the appropriate ethical and privacy reviews were finalized [56]. Key datasets were transferred to the researchers under the Information Sharing Agreement and ethical approval was acquired through the Institutional Research Ethics Boards of the participating universities. In an attempt to protect the identity of individual patients and communities, data were de-identified and analyses were performed to examine patterns of access to PHC services.

### Measures

The main *dependent variable* for this study is the change (yes/no) in BC hospitalization rates for all residents from 1994 to 2010 for mental health-related ACSC. We measure the change in hospitalization rates three different ways: admission, length of stay and 30-day readmission rates. We focused on psychotic disorders (major mental health disorders): ICD-9-CM diagnoses codes 295–299, which include schizophrenic disorders, paranoid conditions and major depressions. These disorders are typically chronic or persistently recurrent, are associated with serious social and occupational disability, and can be reliably identified in administrative data [57]. This category of disorder has been termed serious, major, long-term or chronic in other investigations [32, 58, 59]. We did not select addiction-related hospitalizations because the data are mostly incomplete and unreliable.

The International Classification of Diseases (ICD) codes for mood disorders and schizophrenia were used to detect admissions for these conditions. We used three different measures of hospitalization for mental health-related ACSC: (1) rates of episodic hospital care, (2) rates of length of stay and (3) 30-day readmission rates (see Table 2). An episode of hospital care was defined as an initial hospitalization plus any additional hospitalizations occurring within 24 h of discharge, to capture transfers from one hospital to another. The length of stay was calculated for the total episode of hospital care.

**Table 2:** Definition of dependent variables

Measure of Hospitalization	Definition of Hospitalization Measure
Rates of episodes of hospital care	The discrete number of hospitalization episodes from admission to discharge. Hospitalizations were treated as a single episode when readmission to another hospital occurred within one day, to account for transfers from one hospital to another.
Rates of length of stay per admission	An average of the number of days in hospital for each episode of care.
Readmission rates to acute care	Readmission within 30 days of discharge from the initial admission.

The main *independent variable* for this study focuses on local access to PHC services. We developed a database of First Nations communities in BC that shows the level of PHC available in their community, based on classifications outlined in Table 1. The table shows that only 10 First Nations communities in BC have access to a full complement of PHC services provided by the Nursing station model of care. The remaining communities are resourced with 47 Health Stations offering part-time non-resident screening and prevention services, and 12 Health Centres offering public health programs 5 days a week or less that primarily focus on health education and screening. For 56 communities no facilities are available on reserve, with the expectation that individuals will access services off reserve in a nearby provincial facility. The small number of communities with a *health office* (*n* = 3) were excluded from analysis. The impact on our overall sample was negligible as these communities are very small. While we are using broad categories for on-reserve PHC, which glosses over the variability of services delivered in each community, we believe that this methodology, which was developed with the FNHA, is the most pragmatic and appropriate method to bring evidence to policy and resourcing applications.

We employed a multi-level model-based approach using a generalized estimating equations (GEE) method of parameter estimation to predicate change over time in hospitalization rates, readmission rates, and length of stay for mental health-related ACSC and to detect statistically significant differences between groups, using the statistical package SAS. GEE are used as a method for analyzing correlated longitudinal data. This data has measurements (hospitalization) taken over time on subjects that share common characteristics (age group, sex, SES) living in communities with similar characteristics (access to care at the community level). One may expect the outcomes for subjects of similar age, sex, SES and community to be correlated over time.

The GEE method takes into account the correlated structure of the data and allows for valid hypothesis testing results [27, 60]. We used negative binomial regression models, which better fit data that is over-dispersed. We modeled the rates using an offset = log(population) to determine whether access to different levels of care in First Nations communities is associated with a change in hospitalization rates for mental health related ACSC. We also investigated whether the rates of hospitalizations for mental health-related ACSC differ by geography (on reserve, off reserve and all BC). The type of health facility provided in each First Nation did not change over the period of study, allowing us to reliably examine hospitalizations as a function of access to care. All models reported on are provided in Appendix. To allow for comparison, the analysis was adjusted for age, sex, and socio-economic status (SES).

We recognize statistical challenges in developing our models. Most individuals in any 1 year will not be hospitalized, and examining small communities over one-year periods will not produce stable mean estimates for comparison. Thus, a negative binomial distribution instead of normality was postulated. In addition, and because examining small communities over one-year periods will not produce stable estimates, we aggregated 5 years of data to increase the number of episodes of care, and used rolling time periods to mitigate the instability of smaller sample sizes [27].

## Results

Between 1994 and 2010, we identified a total of 50,825 hospitalizations for mental health related ACSC among First Nations living on or adjacent to a reserve (52% males), compared to 45,983 hospitalizations for First Nations living off reserve (64% males). Table 3 shows the demographic distribution of the population included in this study.

**Table 3:** Population by sex and period of data

Years and gender	Population FN on (and adjacent to a) reserve	Population FN off reserve	Population other BC	Population All BC
1994 Male	26,416	21,093	1,784,732	1,832,241
1994 Female	24,409	24,890	1,794,535	1,843,834
2010 Male	31,617	32,265	2,155,816	2,219,698
2010 Female	29,530	36,301	2,180,395	2,246,226

**Fig. 1:**
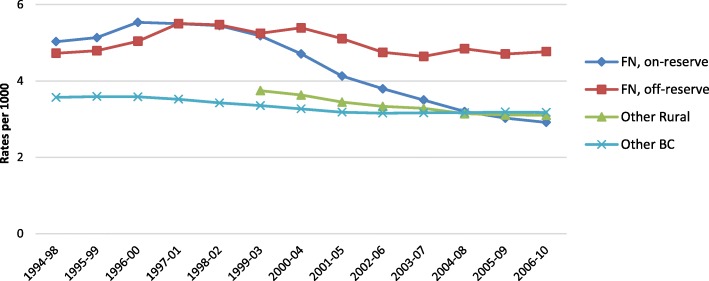
Adjusted rates of hospitalizations for mental health ACSC for First Nations compared to other BC residents (per 1000 population)

Figure 1 shows that the rate of episodes of hospital care for First Nations peoples living on reserve (% change 3.99, *p* < 0.0001) and for other BC residents (% change 0.58, *p* = 0.048) decreased over time (1994–2010). This was not the case for First Nations living off reserve. In addition, First Nations peoples living off reserve had significantly higher rates of hospitalization for mental health-related ACSC compared to others living in BC. As for people living on reserve, the rates were higher compared to other BC residents until 2008, when it began to drop.

**Fig. 2:**
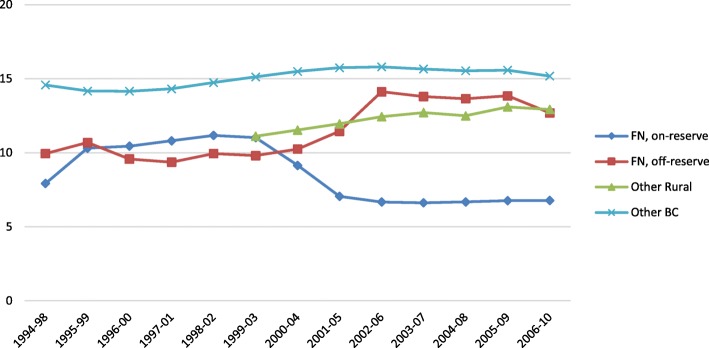
Adjusted rates of LOS/admission for mental health ACSC hospitalizations, for First Nations compared to other BC residents (per 1000 population)

Figure 2 shows that the rates of length of stay in hospital for First Nations living off reserve increased (% change 2.6, *p* = 0.0009) over time. The adjusted length of stay is longer for all other BC residents: on average, people living in BC stay an additional 4–6 days longer compared to First Nations peoples living on and off reserve. This is likely an artifact of geography, since urban residents (a majority of BC non-Indigenous peoples live in urban environments) are more likely to be hospitalized at a higher threshold of acuity than rural/remote community residents, because of the proximity of care should their condition escalate.

**Fig. 3:**
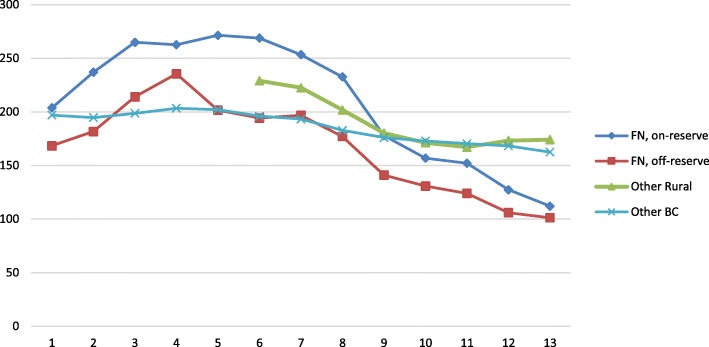
Adjusted readmission rates to acute care for mental health ACSC hospitalizations, for First Nations compared to other BC Residents (per 1000 population)

Figure 3 shows that rates of 30-day readmission have been decreasing for First Nations living on reserve (% change 4.25, *p* < 0.0001), off reserve (% change 3.80, *p* = 0.0006) and for all other BC residents (1.67, *p* < 0.0001). Interestingly, readmission rates for First Nations (on and off reserve) fell below the provincial average after 2002–06.

**Fig. 4:**
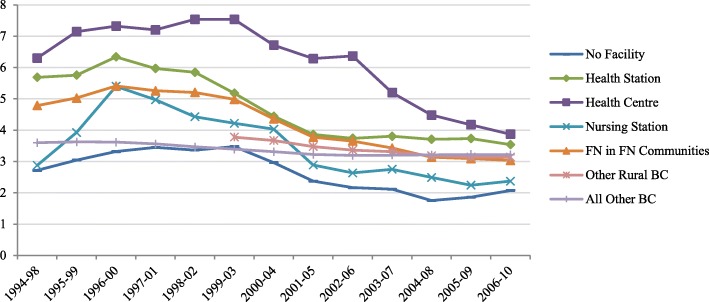
Adjusted rates of hospitalization for mental health ACSC, based on access to facility type (per 1000 population)

Figure 4 shows the rates of hospitalization for mental disorders for First Nations living on reserve, based on local access to health services. While we acknowledge that community differences go beyond access to PHC, findings show that First Nations communities served by nursing stations have the lowest rates of admission for mental health-related ACSC, suggesting that local access to a broader complement of PHC services designed by and for the community yields better mental health outcomes for First Nations. In contrast, communities served by health centres have the highest rates of hospitalization for mental disorders, suggesting poorer access to needed services. In terms of trends, communities served by nursing stations also experienced a significant decrease in hospitalization rates for mental health conditions over time (% change 6.05, *p* = 0.0014). Communities served by health centres or health stations experienced a more modest drop (4.13, *p* = 0.0041; 3.56, *p* = 0.0039 respectively). We report no significant trend for communities located close to a provincial point of care (those with no facility on reserve), but note that they show the lowest rates of hospitalization for mental health conditions. Rates of hospitalization are generally lower for individuals living at close proximity to mental health facilities, compared to individuals from more remote settings for the same level of acuity, because care can more readily be accessed on an outpatient basis. However, this does not fully explain the high rates of hospitalization documented for First Nations living off reserve, who would have similar access. In this case, it may be that living on reserve with First Nations peers can be a protective factor.

### Limitations

A major limitation to our analysis is the exclusion of comparing geographical differences among non-First Nations BC residents. We understand that communities located within the boundaries of metropolitan centres have relatively increased opportunities for socioeconomic mobility compared to rural and remote regions of the provinces with comparably limited opportunities. However, complete data were not available for the entire period under analysis, therefore comparisons could be misleading. As discussed in the Conceptual Framework section of this article, major national self-governance policy changes in First Nations in BC health occurred just after the time period under investigation, and so the chosen analysis is of greater interest to the study, as it offers a baseline for the First Nations Health Authority. It can also indicate areas where FNIHB may need to invest in all other provinces, where the former system remains largely intact. Secondly, the use of facility-type designations (nursing stations, health centres, health office) does not account for the variability of services across communities nor assess the performance of specific services, local practices, and informal or traditional healing services delivered outside the scope of practice. Hence findings are limited from interpreting the specific pathways through which preventable hospitalization rates are produced. There may not be agreement on what mental health conditions are considered ACSC. Nevertheless, relating findings to facility designations, which is associated with specific levels of funding and service delivery, can provide useful information for decision-making. Lastly, Kirmayer and colleagues [60] showed that service utilization studies typically capture a low-end estimate of the actual rate of distress in a community, which our study is susceptible to encounter. However, these authors also suggested that a delay in seeking care might eventually translate into higher service use, especially hospitalization.

## Discussion

Our study intended to elucidate the relationship between on-reserve access to PHC services and rates of hospitalization for mental health-related ACSC among First Nations in BC, Canada. Results from this study demonstrate that during the period of 1994–2010 hospitalization rates for mental health-related ACSC decreased for on-reserve communities and leveled off with all BC residents, signaling a likely improvement in access to primary MHC services (Fig. 1). Communities accessing nursing stations had lower rates of hospitalizations for mental health-related ACSC compared to communities accessing other facility types (Fig. 4). However, other facilities offering limited prevention and screening services constitute 92% (115 of 125, excluding Health Offices) of First Nations communities in BC. The findings build on similar conclusions drawn from a Manitoba study by Lavoie and colleagues [27] which measured local access to PHC services and non-specific ACSC (which excluded mental health conditions). Nursing stations appear to provide a more optimal complement of PHC associated with a reduction in ACSC rates when looking at on-reserve comparisons. We reiterate the conclusions drawn from the Manitoba study on the importance to invest in PHC on reserve and recognize the value of nurses working with an expanded scope of practice [27].

In contrast, even though the rates of mental health-related hospitalizations for ACSC have decreased for off-reserve First Nations over time, they remain significantly higher than other BC residents, who disproportionately make up urban populations. As stated above, this may be a geographical artifact. It is generally the case that urban populations tend to be hospitalized at higher levels of acuity and only when outpatient care is no longer possible. We therefore would expect urban populations to be hospitalized for longer periods of time than rural/remote populations. Figure 2 shows that the length of stay for on- and off-reserve First Nations remains lower than other BC residents, which is consistent with expectations *if* hospitalizations are indeed for lower levels of acuity. Finally, readmission rates (Fig. 3) have decreased dramatically for both on- and off-reserve First Nations, which may signify improvements over time in the appropriateness of care being received. Alternatively, this may be the result of distrust in the effectiveness of hospitalization.

Whereas discriminatory practices occur at an interpersonal level, they also function structurally through policy and service delivery to create culturally unsafe environments for First Nations health by undermining holistic, responsive approaches to care [61, 62]. On-reserve MHC funded through FNIHB is limited and fragmented with services primarily offered in the form of emergency mental health counselling. These administratively onerous services are available to either individuals in crisis situations by providing short-term mental health interventions, or former Indian Residential School students and their families. The program allows a patient up to 20 one-hour counselling sessions per 12 months, often requiring the patient to travel to a provincial facility. For non-crisis cases, patients are expected to access MHC through a provincial facility or community-based service, largely unavailable on reserve [44]. Furthermore, research repeatedly shows that mental disorders and substance abuse often present as co-morbidities within a context of historical trauma [11], yet service responses have been poorly integrated. Government policies have been unable to reflect the multifaceted and shifting needs of communities by upholding narrowly defined proposal-driven funding toward biomedical treatment models [52, 63–65].

Although an allowance subsidizes patients to travel off reserve, medical transportation is constrained by increasing demand, cost containment, uneven coverage and at times unresponsiveness to the needs of First Nations seeking care [66]. While this may have changed since the creation of the FNHA, this was definitely the case during the period under study. While mobility between reserve or rural communities and urban centres has been emphasized in the health literature [66, 67], a study by Snyder and Wilson [68] highlighted the challenges individuals permanently residing off reserve may equally experience. Although their study participants were recruited through service organizations (hence already had higher degrees of access to services), the study raised questions on how intra-community relocation among isolated urban service users may disconnect them from accessing care. Notwithstanding the limited availability of MHC provisions and medical transportation services, service access unravels in light of jurisdictional ambiguities between federal and provincial governments and their responsibility over First Nations health, which have been widely documented in the literature as a barrier to access health care [66, 69–74]. Solutions may require improving access to culturally safe, trauma-informed mental wellness services designed to meet the specific needs of First Nations people living in urban areas.

A key policy option to respond to the geographical and jurisdictional constraints experienced in rural and remote localities might be the expansion of telemental health (TMH). TMH involves the use of information and communications technology (e.g. telephone or videoconferencing) to support the delivery of MHC services to communities unable to physically access them [75]. Although the effectiveness of TMH has not been thoroughly assessed in relation to health outcomes, it is shown to increase access to services, client satisfaction, decrease costs, and facilitate networking [53, 76]. However, challenges with implementing telehealth have been identified in First Nations contexts, including infrastructural development, privacy protection, supporting technical capacity, perceived usefulness by service providers, and cultural values conflicting with technological approaches [52, 77]. While BC offers TMH to on-reserve First Nations, these services are limited and vary across communities, depending on the availability of providers, community infrastructure and other factors [52].

As seen in Figs. 1 and 2, during 2001–05 to 2002–06, we observed a dramatic change in rates of hospitalization for mental health-related ACSC for all measures, which occurred during the deinstitutionalization of psychiatric services. As in many other countries, reports from BC have illustrated the deleterious effects of deinstitutionalizing MHC from a psychiatric to a community-based setting [46, 78].

In BC, decreased funding toward psychiatric institutions was not matched with increased funding for community care. Services were primarily responsive to extreme cases, often for prematurely discharged low-risk patients. A continuum of care integrating social assistance, housing and addictions supports was mostly absent [79, 80]. First Nations programs did not receive additional resources to develop enhanced community-based mental health services. Thus the higher rates of hospitalization for mental health-related ACSC for those First Nations living off reserve may reflect a lack of access to culturally appropriate community-based mental health services for First Nations.

## Conclusion

This study is one of a few recent configurations by the authors to quantitatively illuminate the relationship between national policies informing First Nations’ access to health services and community health outcomes. Greater investments in the provision of culturally safe and informed care and telemental health services could improve outcomes. Although increased investments in primary MHC are paramount, investments should develop within a wider context of addressing social determinants impacting the mental health of Indigenous peoples, including inter-generational trauma, addictions, racism, poverty, housing, child welfare and incarceration [81, 82]. Addressing unmet mental health needs requires moving beyond access to health care services, and equally emphasizing and committing research and resources toward prevention and the sociopolitical factors shaping First Nations mental health. As other national and international jurisdictions look to the findings from this study and BC as a model of health system transformation [83], we hope the information we report on will contribute to the gaps in knowledge obstructing our ability to envision the equitable financing and delivery of primary MHC for Indigenous and rural populations, keeping in mind the unique context of relationships between First Nations in BC and in Canada.

## Appendix

**Table 4:** Summary of model,mental health ACSC admissions

Parameter	Estimate	Standard Error	Lower Conf Level	Upper Conf Level	Z	Pr > |Z|	Exp (Est)
FN on reserve	0.4182	0.0949	0.2321	0.6043	4.40	<.0001	1.52
FN off reserve	0.2575	0.0974	0.0666	0.4484	2.64	0.0082	1.29
Other BC	Reference						
Rolling 5 year average	− 0.0067	0.0035	− 0.0136	0.0002	−1.91	0.0556	0.99
Female	0.4887	0.0891	0.3140	0.6634	5.48	<.0001	1.63
Male	Reference						
Age group 0–14 yrs	−1.4105	0.2155	−1.8329	−0.9882	−6.55	<.0001	0.24
Age group 15–24 yrs	0.6661	0.1947	0.2845	1.0478	3.42	0.0006	1.95
Age group 25–34 yrs	0.8246	0.2070	0.4189	1.2302	3.98	<.0001	2.28
Age group 35–44 yrs	0.6093	0.1971	0.2230	0.9956	3.09	0.0020	1.84
Age group 45–54 yrs	0.5167	0.2037	0.1174	0.9160	2.54	0.0112	1.68
Age group 55–64 yrs	−0.1235	0.2158	−0.5465	0.2995	−0.57	0.5671	0.88
Age group 65–74 yrs	Reference						
Mean SES	−0.0005	0.0164	−0.0327	0.0316	−0.03	0.9740	1.00
Rolling 5 yr. average, FN on reserve	−0.0435	0.0084	−0.0599	−0.0271	−5.19	<.0001	0.96
Rolling 5 yr. average, FN off reserve	0.0063	0.0064	−0.0063	0.0189	0.98	0.3252	1.01
Rolling 5 yr. average, other BC	Reference						

**Table 5:** Summary of model, mental health LOS per admission

Parameter	Estimate	Standard Error	Lower Conf Level	Upper Conf Level	Z	Pr > |Z|	Exp (Est)
FN on reserve	−0.5326	0.1128	−0.7537	− 0.3115	−4.72	<.0001	0.59
FN off reserve	−0.4149	0.0909	−0.5930	−0.2367	−4.57	<.0001	0.66
Other BC	Reference						
Rolling 5 year average	0.0048	0.0030	−0.0011	0.0108	1.60	0.1099	1.00
Female	−0.2320	0.0886	−0.4057	− 0.0584	− 2.62	0.0088	0.79
Male	Reference						
Age group 0–14 yrs	− 0.5722	0.2846	−1.1301	− 0.0143	− 2.01	0.0444	0.56
Age group 15–24 yrs	− 0.6516	0.2764	− 1.1934	− 0.1097	− 2.36	0.0184	0.52
Age group 25–34 yrs	−0.8168	0.2903	− 1.3858	− 0.2477	−2.81	0.0049	0.44
Age group 35–44 yrs	−0.8770	0.2800	−1.4257	−0.3283	−3.13	0.0017	0.42
Age group 45–54 yrs	−0.8713	0.2823	−1.4246	−0.3180	−3.09	0.0020	0.42
Age group 55–64 yrs	−0.4877	0.2981	−1.0719	0.0965	−1.64	0.1018	0.61
Age group 65–74 yrs	Reference						
Mean SES	−0.0246	0.0169	−0.0576	0.0084	−1.46	0.1446	
Rolling 5 yr. average, FN on reserve	−0.0027	0.0095	−0.0214	0.0159	−0.29	0.7734	1.00
Rolling 5 yr. average, FN off reserve	0.0139	0.0053	0.0036	0.0242	2.65	0.0081	1.01
Rolling 5 yr. average, other BC	Reference						

**Table 6:** Summary of model, readmissions after mental health ACSC admissions

Parameter	Estimate	Standard Error	Lower Conf Level	Upper Conf Level	Z	Pr > |Z|	Exp (Estimate)
FN on reserve	0.2755	0.1344	0.0120	0.5390	2.05	0.0404	1.32
FN off reserve	0.0588	0.1406	−0.2167	0.3343	0.42	0.6758	1.06
Other BC	Reference						
Rolling 5 year average	−0.0144	0.0038	−0.0219	− 0.0069	−3.75	0.0002	0.99
Female	0.0533	0.1475	−0.2358	0.3424	0.36	0.7180	1.05
Male	Reference						
Age group 0–14 yrs	0.2436	0.5190	−0.7736	1.2609	0.47	0.6388	1.28
Age group 15–24 yrs	0.5148	0.5197	−0.5037	1.5334	0.99	0.3218	1.67
Age group 25–34 yrs	0.5775	0.5309	−0.4630	1.6180	1.09	0.2767	1.78
Age group 35–44 yrs	0.4613	0.5151	−0.5482	1.4708	0.90	0.3705	1.59
Age group 45–54 yrs	0.7043	0.5220	−0.3189	1.7275	1.35	0.1773	2.02
Age group 55–64 yrs	0.3530	0.5338	−0.6931	1.3992	0.66	0.5083	1.42
Age group 65–74 yrs	Reference						
Mean SES	−0.0134	0.0296	−0.0715	0.0447	−0.45	0.6506	0.99
Rolling 5 yr. average, FN on reserve	−0.0247	0.0120	−0.0482	−0.0012	−2.06	0.0396	0.98
Rolling 5 yr. average, FN off reserve	−0.0242	0.0134	−0.0504	0.0021	−1.80	0.0712	0.98
Rolling 5 yr. average, other BC	Reference						

**Table 7:** for model for Mental Health ACSC Admissions by Facility type (access to local services)

Parameter	Estimate	Standard Error	LCL	UCL	Z	Pr > |Z|	Exp (Estimate)
No facility	−0.2134	0.1426	−0.4929	0.0660	−1.50	0.1344	0.81
Health Station	0.3239	0.1390	0.0516	0.5963	2.33	0.0197	1.38
Health Centre	0.7435	0.1822	0.3863	1.1006	4.08	<.0001	2.10
Nursing Station	0.3314	0.1889	−0.0388	0.7017	1.75	0.0793	1.39
Other BC	Reference						
Rolling 5 year average	−0.0045	0.0033	−0.0109	0.0019	−1.37	0.1697	1.00
Female	0.4017	0.0923	0.2207	0.5827	4.35	<.0001	1.49
Male	Reference						
Age group 0–14 yrs	−1.5142	0.2021	−1.9103	− 1.1182	−7.49	<.0001	0.22
Age group 15–24 yrs	0.6531	0.1825	0.2953	1.0108	3.58	0.0003	1.92
Age group 25–34 yrs	0.7583	0.1951	0.3760	1.1406	3.89	0.0001	2.13
Age group 35–44 yrs	0.6526	0.2012	0.2583	1.0470	3.24	0.0012	1.92
Age group 45–54 yrs	0.4909	0.1943	0.1100	0.8717	2.53	0.0115	1.63
Age group 55–64 yrs	−0.0586	0.2062	−0.4629	0.3456	−0.28	0.7762	0.94
Age group 65–74 yrs	Reference						
Mean SES	−0.0122	0.0183	−0.0481	0.0236	−0.67	0.5038	0.99
Rolling 5 yr. average, no facility	−0.0115	0.0139	−0.0387	0.0157	−0.83	0.4058	0.99
Rolling 5 yr. average, Health Station	−0.0324	0.0133	−0.0585	−0.0064	−2.44	0.0146	0.97
Rolling 5 yr. average, Health Centre	−0.0401	0.0156	−0.0706	−0.0096	−2.57	0.0101	0.96
Rolling 5 yr. average, Nursing station	−0.0581	0.0195	−0.0963	−0.0200	−2.99	0.0028	0.94
Rolling 5 yr. average, other BC	Reference						

## Abbreviations

ACSC: Ambulatory care sensitive conditions
BC: British Columbia
DAD: Discharge Abstract Database
FNHA: First Nations Health Authority
FNIHB: First Nations and Inuit Health Branch of Health Canada
GEE: Generalized estimating equations
ICD: International Classification of Diseases
IRS RHSP: Indian Residential Schools Resolution Health Support Program
MB: Manitoba
MHC: Mental Health Care
MSP: Medical Services Plan
NAYSPS: National Aboriginal Youth Suicide Prevention Strategy
NNADAP: National Native Alcohol and Drug Abuse Program
PHC: Primary health care
STCIMHC: Short-Term Crisis Intervention Mental Health Counselling
TMH: Telemental health
